# Heat and Mass Transfer in Unsteady Rotating Fluid Flow with Binary Chemical Reaction and Activation Energy

**DOI:** 10.1371/journal.pone.0107622

**Published:** 2014-09-24

**Authors:** Faiz G. Awad, Sandile Motsa, Melusi Khumalo

**Affiliations:** 1 Department of Pure & Applied Mathematics, University of Johannesburg, Auckland Park, Johannesburg, South Africa; 2 School of Mathematics, Statistics and Computer Science, University of KwaZulu-Natal, Scottsville, Pietermaritzburg, South Africa; North China Electric Power University, China

## Abstract

In this study, the Spectral Relaxation Method (SRM) is used to solve the coupled highly nonlinear system of partial differential equations due to an unsteady flow over a stretching surface in an incompressible rotating viscous fluid in presence of binary chemical reaction and Arrhenius activation energy. The velocity, temperature and concentration distributions as well as the skin-friction, heat and mass transfer coefficients have been obtained and discussed for various physical parametric values. The numerical results obtained by (SRM) are then presented graphically and discussed to highlight the physical implications of the simulations.

## Introduction

The study of boundary layer flow and heat transfer inducted by stretching surface has attracted considerable interest due to its wide applications in industrial processes such as the cooling of an infinite metallic plate in a cooling bath, the aerodynamic extrusion of plastic sheets, boundary layer along the material handling conveyers, the boundary layer along a liquid film and condensation processes. The quality of the final product depends on the skin friction coefficient and the rate of heat transfer. One of the earliest studies of the boundary layer flow problem was conducted by Sakiadis [1], [2]. Crane [3] extended this concept to present the problem of the steady two-dimensional boundary layer flow over stretching sheet of elastic flat surface with linear velocity. He demonstrated that the problem was interesting because it possessed a closed form exact solution. Studies have been carried out for the case of the axisymmetric and three-dimensional flow by Brady and Acrivos [4], and Wang [5]. Investigations by, among others, Afzal [6], Prasad et al. [7], Abel and Mahesha [8], Bataller [9], Abel et al. [10], have also provided examples of various aspects of this important field.

Unsteady flows in rotating fluid have numerous uses or potential applications in chemical and geophysical fluid dynamics and mechanical nuclear engineering. Using the Fourier series analysis, Soundalgekar et al. [11] investigated the unsteady rotating flow of incompressible, viscous fluid past an infinite porous plate. The boundary layer flow problem formed in a rotating fluid by oscillating flow over an infinite half-plate has been examined Bergstrom [12]. Abbas et al. [13] studied the unsteady boundary layer MHD flow and heat transfer on a stretching continuous sheet in a viscous incompressible rotating fluid numerically using the Keller-box method. Nazar et al. [14] investigated unsteady flow due to the impulsive starting from rest of a stretching surface in a viscous and incompressible rotating fluid. Zheng et al. [15] studied the unsteady rotating flow of a generalized Maxwell fluid with fractional derivative model between two infinite straight circular cylinders. Using the shooting method Fang [16] studied the problem of the laminar unsteady flow over a stretchable rotating disk with deceleration is investigated. Rashad [17] investigated the unsteady magnetohydrodynamics boundary-layer flow and heat transfer for a viscous laminar incompressible electrically conducting and rotating fluid due to a stretching surface embedded in a saturated porous medium with a temperature-dependent viscosity in the presence of a magnetic field and thermal radiation effects. Nageeb et al. [18] used the Runge-Kutta method based on shooting technique to investigate the unsteady MHD flow and heat transfer of a couple stress fluid over a rotating disk. For the case in which steady flow rotating flow involve the powe-law, very recently, Hajmohammadi et al. [19] developed an analytical solution for two-phase flow betwen two rotating cylinders filed with power law liquid and a micro layer of gas. Moreover Hajmohammadi and Nourazar [20] the problem of heat transfer repercussions thin gas layer in micro cylindrical Couette flows involving power-law liquids.

Many chemically reacting systems involve the species chemical reactions with finite Arrhenius activation energy, with examples occurring in geothermal and oil reservoir engineering. The interactions between mass transport and chemical reactions are generally very complex, and can be observed in the production and consumption of reactant species at different rates both within the fluid and the mass transfer. One of the earliest studies involving the binary chemical reaction in boundary layer flow was published by Bestman [21] who presented an analytical solution using the perturbation method to show the effect of the activation energy in natural convection in a porous medium. Using the Arrhenius activation energy Bestman [22] subsequently investigated radiative heat transfer on the flow of a combustible mixture in a vertical pipe. Makinde et al. [23] studied the effects of 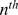 order Arrhenius chemical reaction, thermal radiation, suction/injection and buoyancy forces on unsteady convection of a viscous incompressible fluid past a vertical porous plate numerically. They showed that the effect of the chemical reaction, heat source, and suction or injection is significant at the wall of the wedge on the flow field. A numerical study of the unsteady mixed convection with Dufour and Soret effects past a semi-infinite vertical porous flat plate moving through a binary mixture of chemically reacting fluid was conducted by Makinde and Olanrewaju [24]. The most recent contributions in this area include those of Abdul Maleque [25]–[27], who investigated the effects of chemical reactions with Arrhenius activation Energy on unsteady convection heat and mass transfer boundary layer fluid flow.

This work deals with the effects of chemical reactions with finite Arrhenius activation energy on unsteady rotating fluid flow due to a stretching surface with Binary chemical reaction and activation energy. The governing partial differential equations are solved using the spectral relaxation method (SRM). The SRM is based on simple decoupling and rearrangement of the governing nonlinear equations in a Gauss-Seidel manner. The resulting sequence of equations are integrated using the Chebyshev spectral collocation method. The SRM was introduced in [29] for the solution of the nonlinear ODE system model of von Karman flow of a Reiner-Rivlin fluid. A generalised presentation of the method was later presented in [30] and applied in three ODE based systems of boundary layer flow equations of varying complexity. The method has also been successfully used in the solution of chaotic and hyper-chaotic systems [31], [32] which are defined as systems of ODE initial value problems.

## Mathematical Formulation

Consider the three-dimensional, unsteady flow due to a stretching surface in a rotating fluid. The motion in the fluid is three dimensional. At time 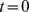, the surface 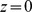 is impulsively stretched in the 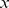 direction in the rotating fluid. The velocity components are assume to be 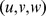 in the direction of the Cartesian axes 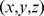, respectively, and the axes is rotating at an angular velocity 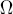 in the 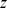 direction. The surface temperature 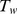 and solute concentration 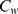 are higher than the ambient values 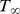 and 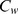, respectively. Assuming a species chemical reaction with finite Arrhenius activation energy, the governing equations for the problem can be written in the form
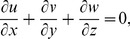
(1)

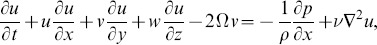
(2)

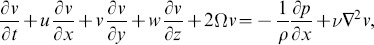
(3)

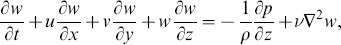
(4)

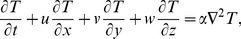
(5)

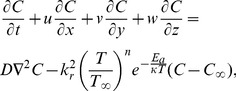
(6)where 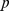 is the pressure, 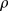 is the density, 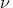 is the kinematic viscosity, 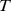 is the fluid temperature, 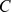 is the solutal concentration, 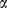 is the thermal diffusivity, 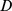 is the solutal diffusivity and 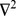 denotes the three-dimensional Laplacian, 
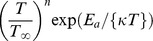
 is the modified Arrhenius function, 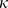 is the Boltzmann constant, 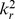 is the chemical reaction rate constant, 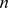 is a unit less constant exponent fitted rate constants typically lie in the range 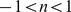. Let the surface be impulsively stretched in the 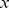 direction such that the initial and boundary conditions are
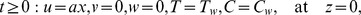


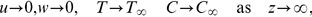
(7)

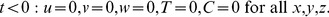
(8)


The following non-dimensional variables are introduced,
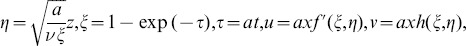


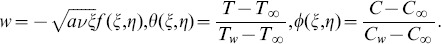
(9)


The governing equations (2) – (5) along with the boundary conditions (7) can be presented as
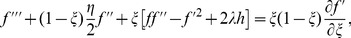
(10)

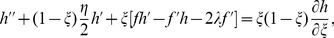
(11)

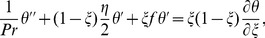
(12)

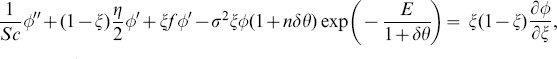
(13)subject to the boundary conditions
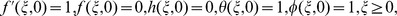


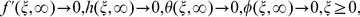
(14)where 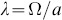 is the rotation rate parameter, 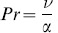 is the Prandtl number, 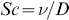 is the Schmidt number, 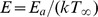 the non-dimensional activation energy, 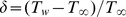 is the temperature relative parameter, 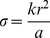 is the dimensionless chemical reaction rate constant.

The non-dimensional skin friction in both 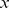 and 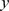 directions, the local Nusselt number, the local Sherwood number are defined in the form
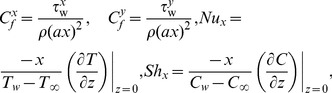
(15)where the wall shear stresses 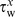, 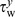, respectively, are given by
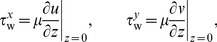
(16)substituting (9) and (16) into (15) it gives
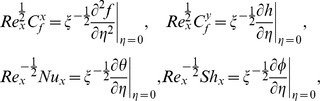
where 
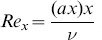
 is the local Reynolds number.

## Numerical Solution

In this section, the spectral relaxation method (SRM) is applied to solve the governing nonlinear PDEs (10 – 13). For the implementation of the spectral collocation method, at a later stage, it is convenient to reduce the order of equation (10) from three to two. To this end, we set 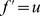, so that equation (10) becomes
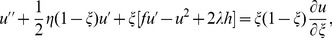
(17)

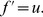
(18)


The spectral relaxation method algorithm uses the idea of the Gauss-Seidel method to decouple the governing systems of equations (10 – 13). From the decoupled equations an iteration scheme is developed by evaluating linear terms in the current iteration level (denoted by 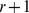) and nonlinear terms in the previous iteration level (denoted by 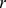). Applying the SRM on (11 – 13) and (17 – 18) gives the following linear partial differential equations;
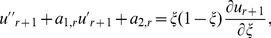
(19)

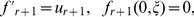
(20)

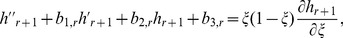
(21)

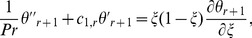
(22)

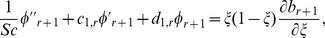
(23)

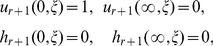
(24)

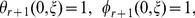
(25)

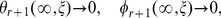
(26)where
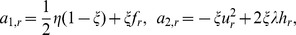


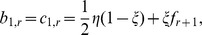


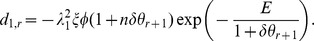



The initial approximation for solving equations (10 – 13) are obtained as the solutions at 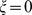. Thus 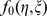, 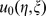, 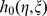, 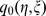 and 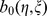 are given by
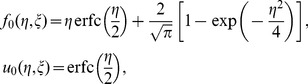
(27)

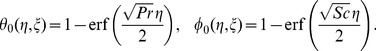
(28)


Starting from given initial approximations (27 – 28), the iteration schemes (19 – 26) can be solved iteratively for 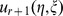, 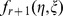, etc, when 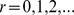. To solve equation (19 – 26) the the linear equations are discretized using the Chebyshev spectral method in the 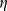-direction and use an implicit finite difference method in the 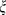-direction. For brevity, the details of the spectral methods are omitted. Interested readers may refer to Refs. [28], [33]. Before applying the spectral method, it is convenient to transform the domain on which the governing equation is defined to the interval [−1,1] where the spectral method can be implemented. For the convenience of the numerical computations, the semi-infinite domain in the space direction is approximated by the truncated domain 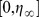, where 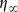 is a finite number selected to be large enough to represent the behaviour of the flow properties when 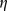 is very large. We use the transformation 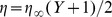 to map the interval 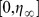 to 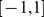. The basic idea behind the spectral collocation method is the introduction of a differentiation matrix 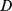 which is used to approximate the derivatives of the unknown variables 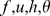 and 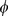 at the collocation points (grid points) as the matrix vector product
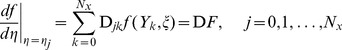
(29)where 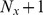 is the number of collocation points, 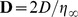, and
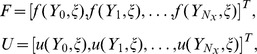


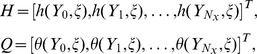


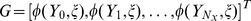
are the vector functions at the collocation points. Higher order derivatives are obtained as powers of 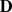, that is
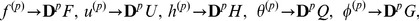
(30)where 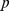 is the order of the derivative. The grid points on 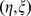 are defined as
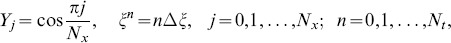
(31)where 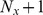, 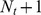 are the total number of grid points in the 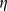 and 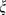-directions respectively, and 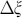 is the spacing in the 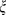-direction. The finite difference scheme is applied with centering about a mid-point halfway between 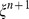 and 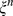. This mid-point is defined as 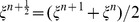. The derivatives with respect with 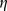 are defined in terms of the Chebyshev differentiation matrices. Applying the centering about 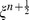 to any function, say 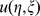 and its associated derivatives we obtain,
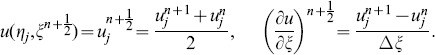
(32)


Thus, applying the spectral collocation method and finite difference approximation on the SRM scheme (19 – 26) gives
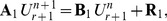
(33)

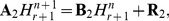
(34)

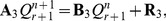
(35)

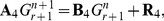
(36)

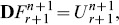
(37)subject to the following boundary and initial conditions
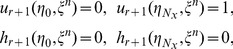
(38)

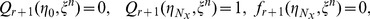
(39)

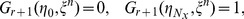
(40)for 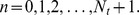 The matrices 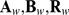 are defined for 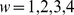 as
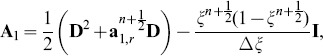


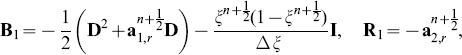


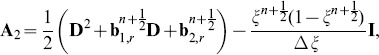


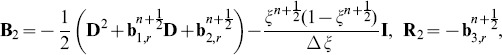


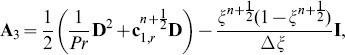


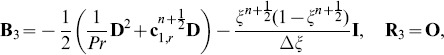


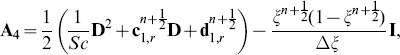


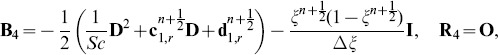
where 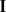 is an 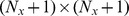 identity matrix and 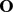 is an 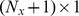 matrix of zeros. The boundary conditions are imposed on the first and last rows of equation each matrix 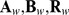. Thus, starting from the initial conditions 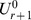, 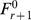, 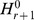, 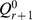, 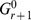 given by equations (27) and (28), the matrix equations (33 – 37) can be solved iteratively, in turn, to give approximate solutions for 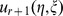, 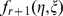, etc, for 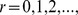 until a solution that converges to within a given accuracy level is obtained.

## Results and Discussion

**Figure 1:**
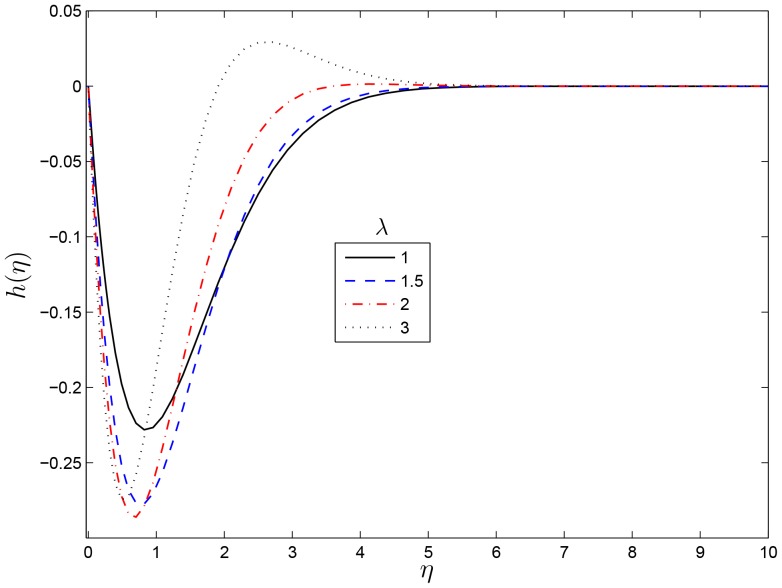
Effect of the rotating parameter 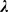 on 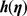 for 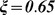, 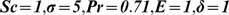 and 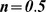.

**Figure 2:**
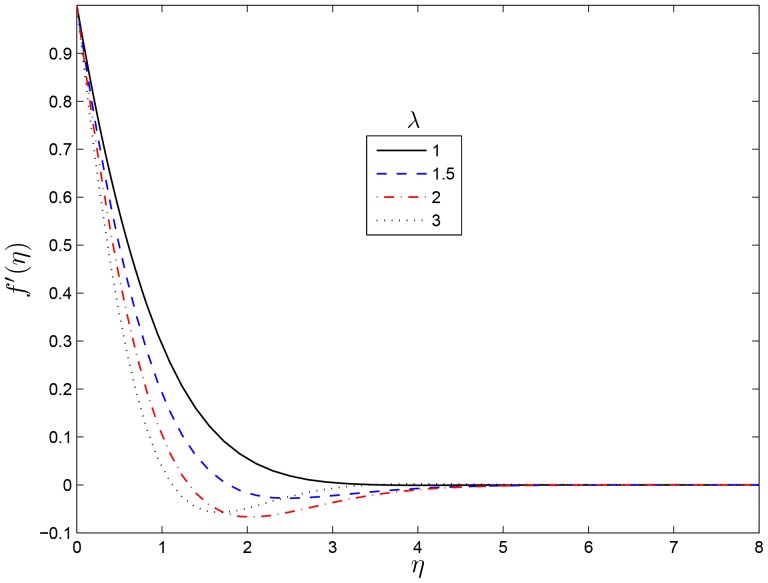
Effect of the rotating parameter 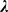 on 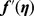 for 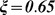, 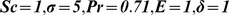 and 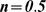.

**Figure 3:**
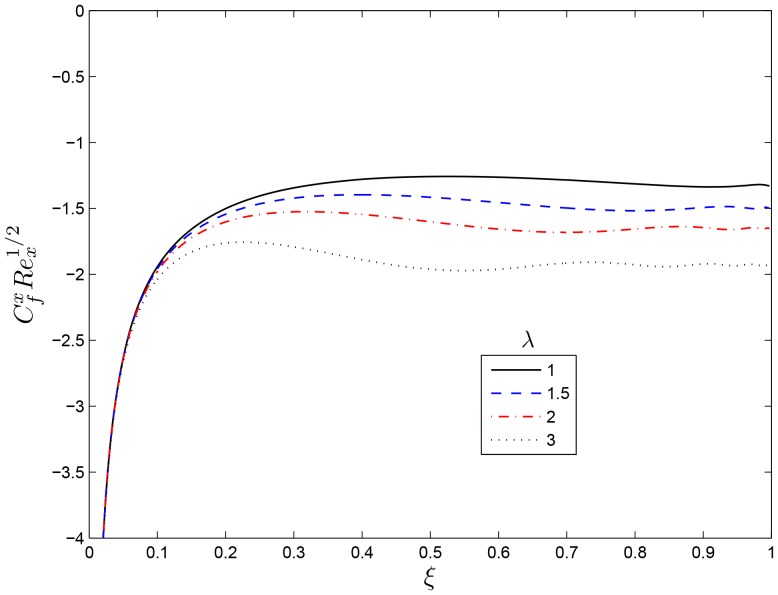
Effect of the rotating parameter 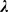 on 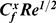 for 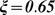, 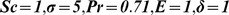 and 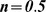.

**Figure 4:**
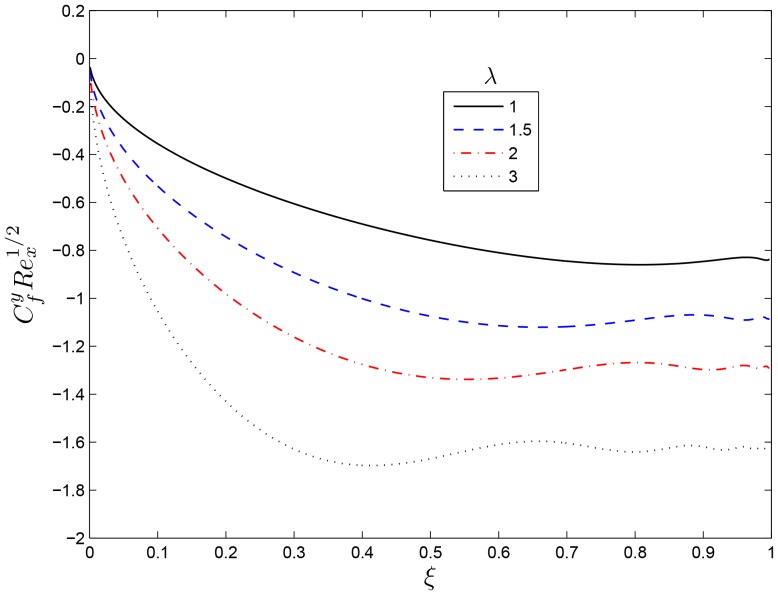
Effect of the rotating parameter 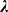 on 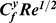 for 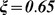, 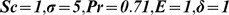 and 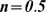.

**Figure 5:**
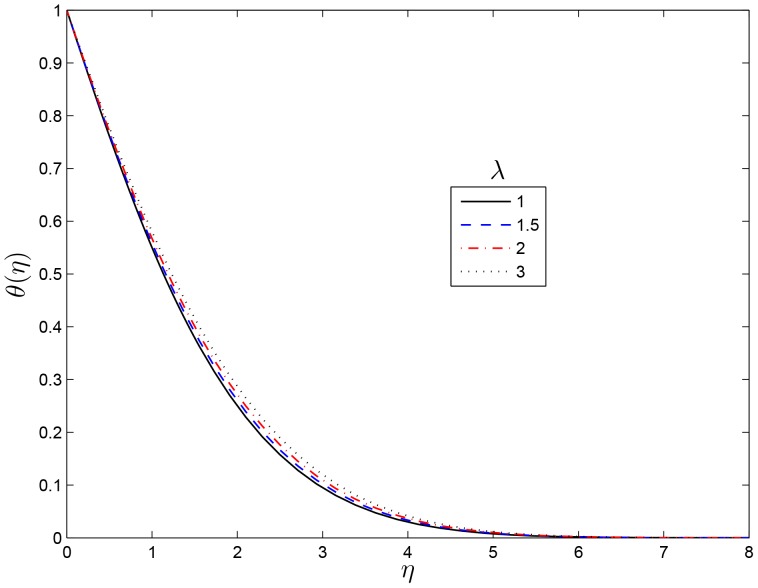
Effect of the rotating parameter 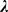 on 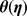 for 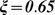, 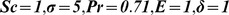 and 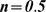.

**Figure 6:**
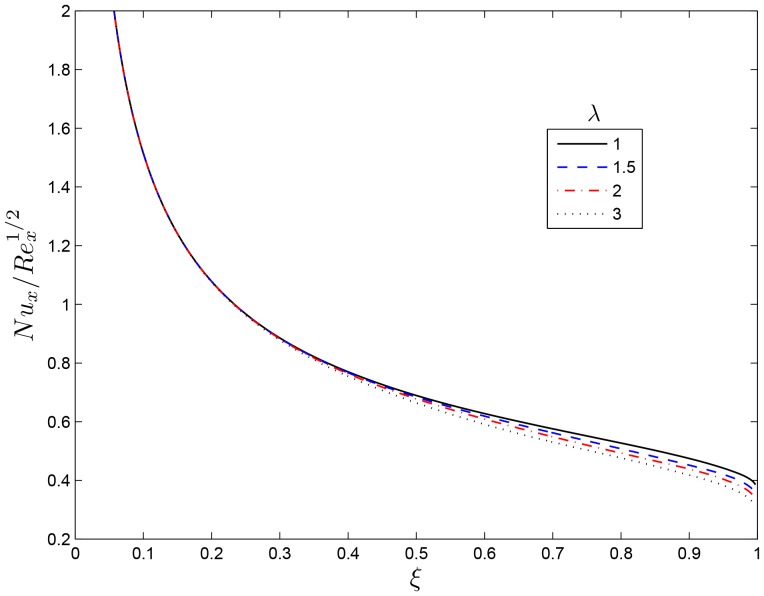
Effect of the rotating parameter 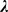 on 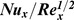 for 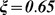, 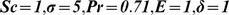 and 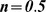.

**Figure 7:**
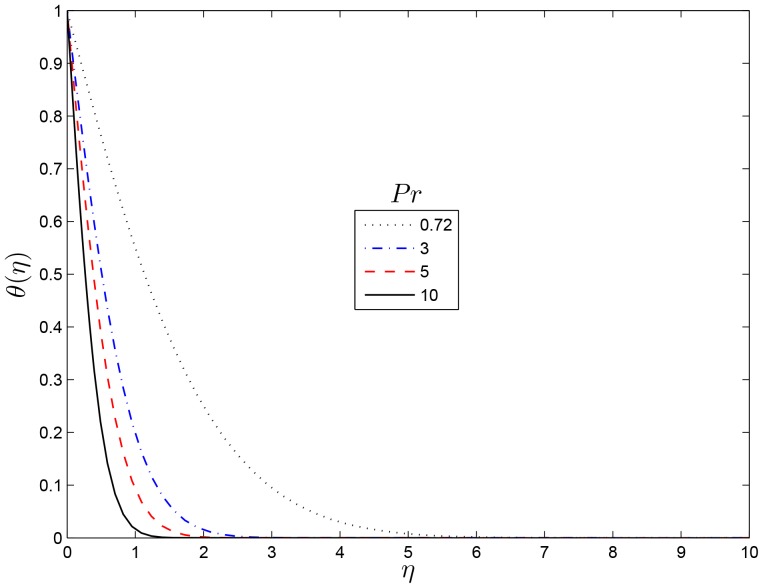
Effect of the rotating parameter 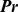 on 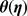 for 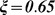, 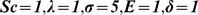 and 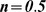.

**Figure 8:**
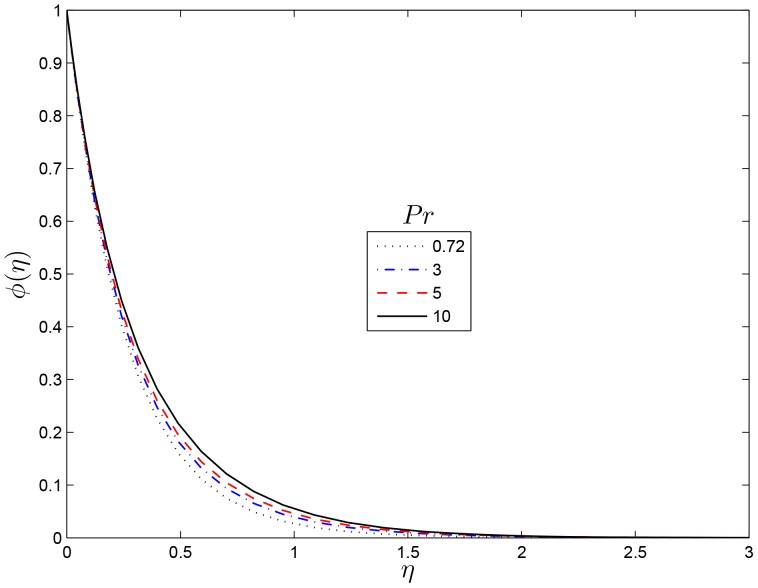
Effect of the rotating parameter 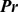 on 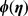 for 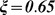, 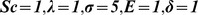 and 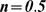.

**Figure 9:**
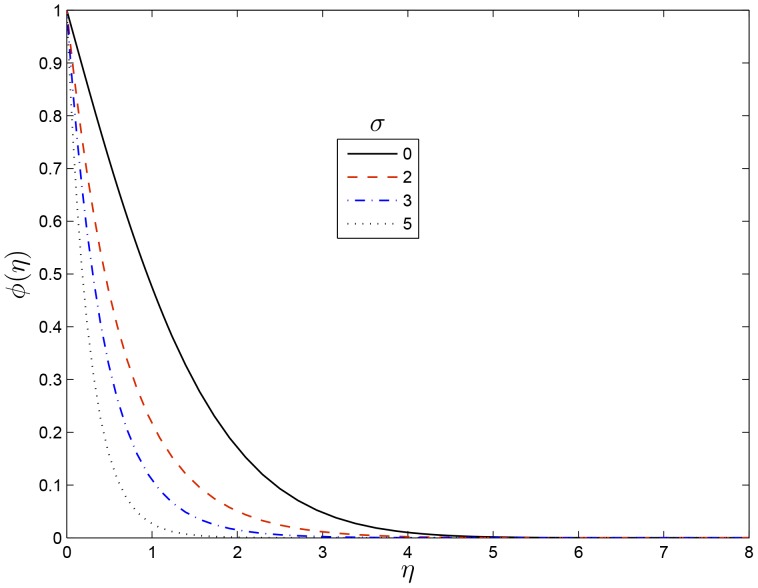
Effect of 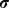 on 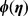 for 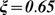, 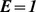, 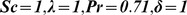 and 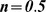.

**Figure 10:**
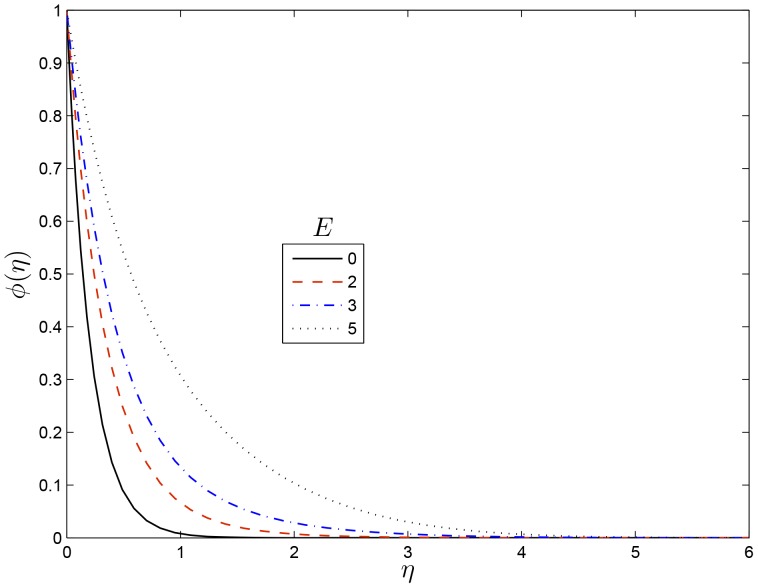
Effect of 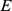 on 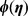 for 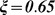, 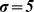, 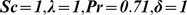 and 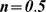.

In order to determine the evolution of the boundary layer flow properties, numerical solutions of the set of governing systems of partial differential equations (10) – (13) along with the boundary conditions (14), were computed using the proposed spectral relaxation method (SRM). Starting from the initial analytical solutions at 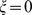 (corresponding to 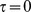), the SRM scheme was used to generate results up to solutions near the steady state values at 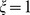 (corresponding to 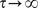). The effect of the governing parameters namely, the rotation rate parameter 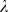, the Schmidt number 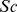, the non-dimensional activation energy 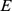, the Prandtl number 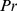, the chemical reaction rate constant 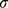, the temperature relative parameter 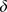 and 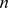 on the flow characteristics as well as the local skin friction, heat and mass transfer coefficients the results are presented graphically in this section. Fig. 1 and Fig. 2 show the variation of the velocity profiles 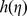 and 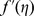, respectively, for different values of 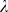. We observe that an increase in the values of 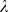 leads to monotonic exponential decay in the velocity profiles for small values and it results in oscillatory decay for a large values of 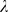. The same results have been reported by Nasar et al. [14] in a related study. Fig. 3 and Fig. 4 show the variation of the skin friction coefficients in the 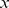 and 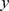 directions respectively for various values of the rotation rate parameter 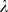. It is observed that 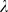 decreases both the skin friction coefficients thus reduces the momentum boundary layers. The effects of the rotation rate parameter 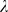 on the temperature profile is shown in Fig. 5. This figure shows that the thermal boundary layer thickness decreases with 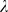, thus an increase in 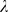 causing a drop in the temperature. Fig. 6 illustrates the variation of the Nusselt number 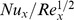 with 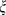 for some values of 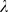. However increases 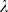 decreases the heat transfer coefficient and the influence of 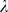 can be obtained beyond 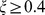 in the heat. The variations of the temperature 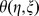 profile with 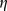 for several values of the Prandtl number 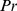 are shown in Fig. 7. It is observed that the thermal boundary layer thickness decrease with an increase in 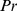. Larger values of Prandtl number corresponds to the weaker thermal diffusivity and thinner boundary layer, hence 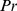 reduces the temperature. Fig. 8 shows concentration distribution for several values Prandtl number. The effect of the Prandtl number is to reduce the mass transfer boundary-layer thickness and so reducing the 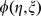. The influence of the chemical reaction rate constant 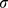 on the concentration profile within the boundary layer is given in Fig. 9. An increase in the 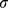 effect reduces the concentration within the thermal boundary layer region. This is because increasing the chemical reaction rate causes a thickening of the mass transfer boundary layer. The effects of the non-dimensional activation energy 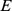 on the concentration profile have been plotted in Fig. 10, it has been notice that increasing the non-dimensional activation energy 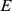 effect increases the concentration boundary layer thinness which enhances the concentration.

**Figure 11:**
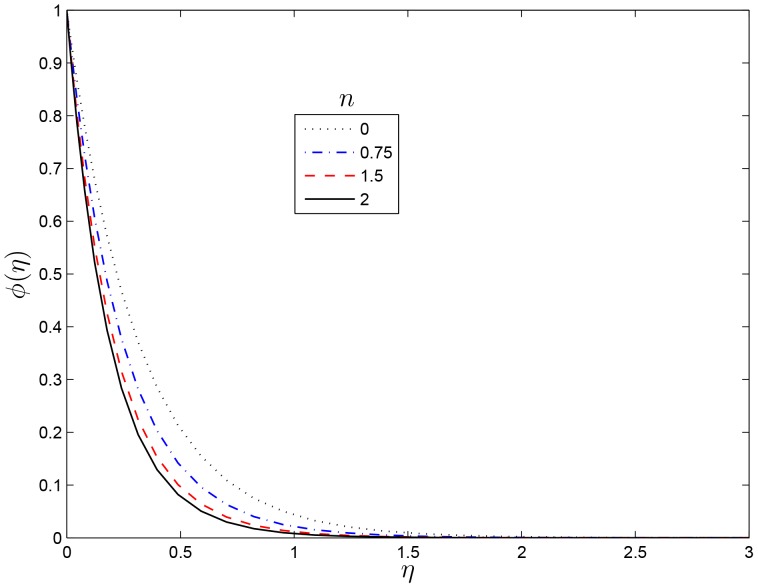
Effect of the rotating parameter 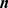 on 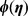 for 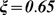, 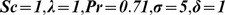 and 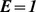.

**Figure 12:**
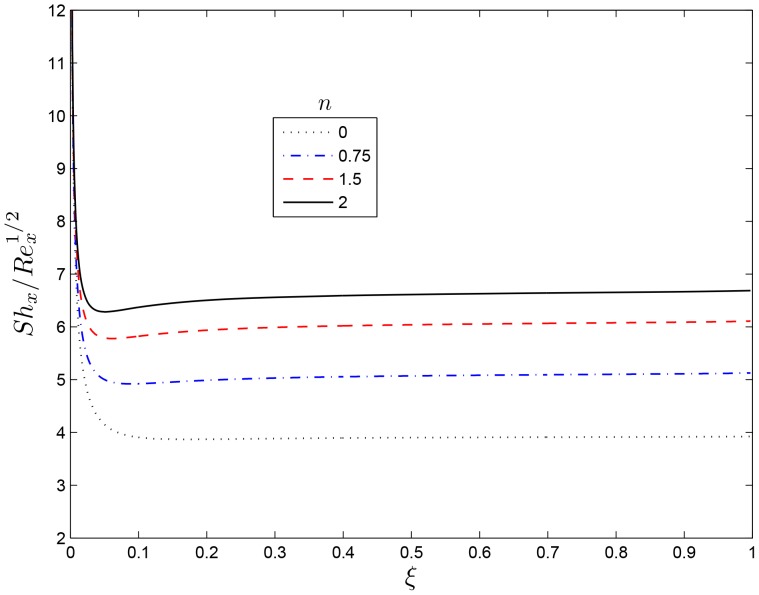
Effect of the rotating parameter 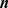 on 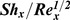 for 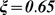, 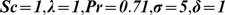 and 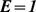.

**Figure 13:**
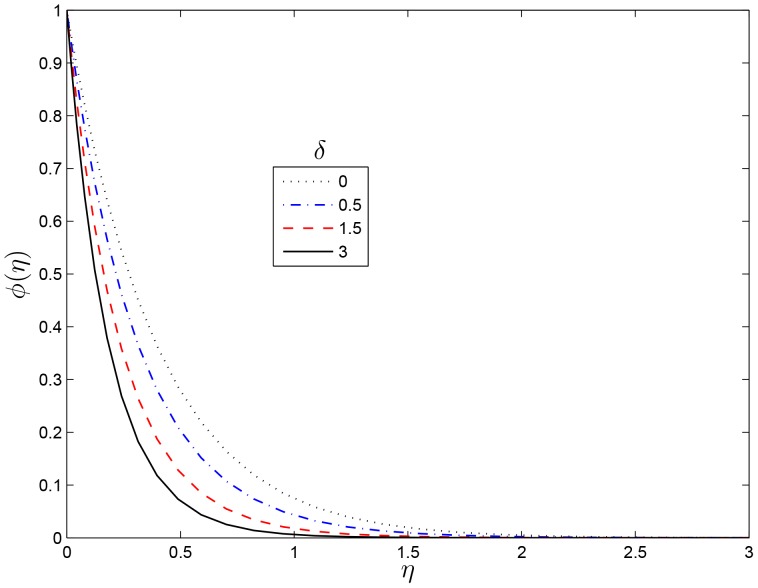
Effect of the rotating parameter 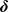 on 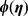 for 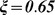, 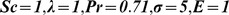 and 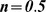.

**Figure 14:**
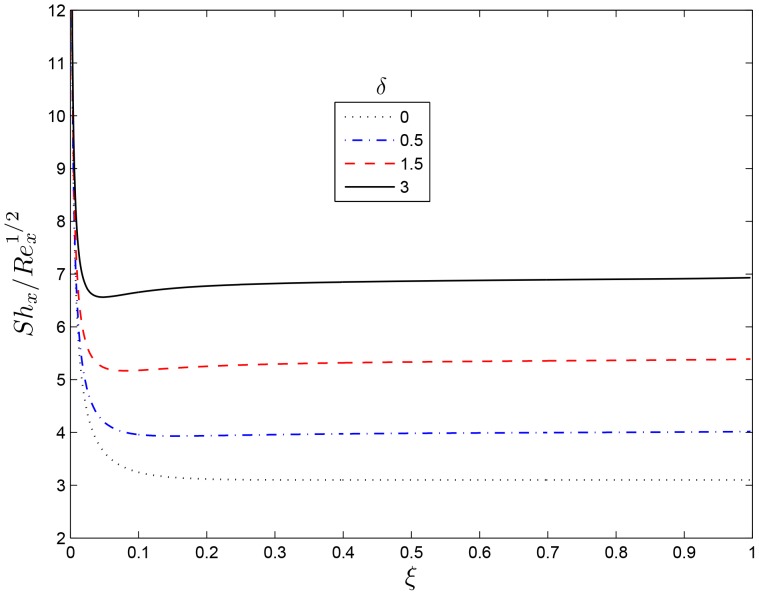
Effect of the rotating parameter 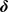 on 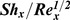 for 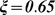, 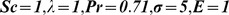 and 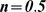.

Fig. 11 shows the effect of increasing the dimensionless exponent fitted rate constant 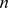 on the concentration profile. It is observed that increasing 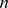 reduces the concentration within the thermal boundary layer leading to an increase in the concentration gradient at the sheet. From Fig. 12 dimensionless exponent fitted rate constant 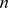 leads to a considerable thinning of the concentration boundary layer, and hence a reduction in mass transfer rate at the sheet wall. Fig. 13 and Fig. 14 depict the variation of the solute concentration and the mass transfer rate 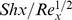 respectively for different values of the temperature relative parameter 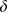. It is evident that as 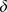 increases, the concentration boundary layer thickness decreases followed by a reduction in both the solute concentration and the mass transfer rate.

## Conclusions

In this investigation, we considered the spectral relaxation method approach to solving an coupled non-linear partial differential equation system that governs the unsteady flow with binary chemical reaction and activation energy due to a stretching surface in a rotating fluid. The effects of the governing parameters namely the rotation rate parameter, the Schmidt number, the non-dimensional activation energy, the Prandtl number, the chemical reaction rate constant, the temperature relative parameter and on the flow characteristics as well as the local skin friction, heat and mass transfer coefficients have been studied. Small values the rotation rate parameter 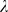 shows a monotonic exponential decay in the velocity profiles and there is oscillatory decay for a large values. Increasing in the non-dimensional activation energy 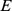 enhances the concentration profile within the boundary layer. The spectral relaxation method used was found to be a very effective method for solving the type of problem considered in this work.
